# Retraction: rs10499194 polymorphism in the tumor necrosis factor-α inducible protein 3 (TNFAIP3) gene is associated with type-1 autoimmune hepatitis risk in Chinese Han population

**DOI:** 10.1371/journal.pone.0195181

**Published:** 2018-03-27

**Authors:** The PLOS ONE Editors

Following publication of this article, concerns were raised about the integrity of the reported work. The authors were unable to provide the underlying raw data for the study or additional methodological information regarding patient recruitment and have stated that most of the information reported in the article had been provided by a scientific service company that assisted with writing the paper.

Owing to this, the *PLOS ONE* Editors were unable to verify the integrity of the content of the article and the source of the data. Furthermore, the authors’ specific contributions to the work are unclear, and the authors do not meet the journal authorship requirement to be accountable for all aspects of the work.

In light of the above concerns, the *PLOS ONE* Editors retract this article.

The authors did not comment on the retraction decision.
